# Metabolic profile of leukemia cells influences treatment efficacy of L-asparaginase

**DOI:** 10.1186/s12885-020-07020-y

**Published:** 2020-06-05

**Authors:** Katerina Hlozkova, Alena Pecinova, Natividad Alquezar-Artieda, David Pajuelo-Reguera, Marketa Simcikova, Lenka Hovorkova, Katerina Rejlova, Marketa Zaliova, Tomas Mracek, Alexandra Kolenova, Jan Stary, Jan Trka, Julia Starkova

**Affiliations:** 1 CLIP – Childhood Leukaemia Investigation Prague, Prague, Czech Republic; 2 Department of Pediatric Hematology and Oncology, Second Faculty of Medicine, Charles University, Prague, Czech Republic; 3 Department of Bioenergetics, Institute of Physiology of the Czech Academy of Sciences, Prague, Czech Republic; 4 University Hospital Motol, Prague, Czech Republic; 5 Department of Pediatric Hematology and Oncology, National Institute of Children’s Diseases and Medical Faculty, Comenius University, Bratislava, Slovakia

**Keywords:** cancer metabolism, L-asparaginase, leukemia, resistance, glycolysis, mitochondrial respiration, fatty acid oxidation, mitochondrial membrane potential

## Abstract

**Background:**

Effectiveness of L-asparaginase administration in acute lymphoblastic leukemia treatment is mirrored in the overall outcome of patients. Generally, leukemia patients differ in their sensitivity to L-asparaginase; however, the mechanism underlying their inter-individual differences is still not fully understood. We have previously shown that L-asparaginase rewires the biosynthetic and bioenergetic pathways of leukemia cells to activate both anti-leukemic and pro-survival processes. Herein, we investigated the relationship between the metabolic profile of leukemia cells and their sensitivity to currently used cytostatic drugs.

**Methods:**

Altogether, 19 leukemia cell lines, primary leukemia cells from 26 patients and 2 healthy controls were used. Glycolytic function and mitochondrial respiration were measured using Seahorse Bioanalyzer. Sensitivity to cytostatics was measured using MTS assay and/or absolute count and flow cytometry. Mitochondrial membrane potential was determined as TMRE fluorescence.

**Results:**

Using cell lines and primary patient samples we characterized the basal metabolic state of cells derived from different leukemia subtypes and assessed their sensitivity to cytostatic drugs. We found that leukemia cells cluster into distinct groups according to their metabolic profile. Lymphoid leukemia cell lines and patients sensitive to L-asparaginase clustered into the low glycolytic cluster. While lymphoid leukemia cells with lower sensitivity to L-asparaginase together with resistant normal mononuclear blood cells gathered into the high glycolytic cluster. Furthermore, we observed a correlation of specific metabolic parameters with the sensitivity to L-asparaginase. Greater ATP-linked respiration and lower basal mitochondrial membrane potential in cells significantly correlated with higher sensitivity to L-asparaginase. No such correlation was found in the other cytostatic drugs tested by us.

**Conclusions:**

These data support that cell metabolism plays a prominent role in the treatment effect of L-asparaginase. Based on these findings, leukemia patients with lower sensitivity to L-asparaginase with no specific genetic characterization could be identified by their metabolic profile.

## Background

Leukemia is the most common malignancy and the second most frequent general cause of childhood death. It is classified as acute lymphoid of the B- or T-lineage being the most prevalent type in children, and as acute myeloid. Although tremendous improvements have been made in the treatment of leukemia in the past few years, there is still a large proportion of patients who do not benefit from the available therapy. The overall survival of pediatric acute lymhoblastic leukemia (ALL) and acute myeloid leukemia (AML) patients treated with the current chemotherapy regimens is above 80 and 70%, respectively [1, 2].

The standardized treatment protocols consist of the same repertoire of cytostatic drugs which have been used for the past decades. They differ in the drug dosage, the time of administration and in the drug combinations. State-of-the-art genomic techniques have enabled the identification of new genetic alterations, which could be targeted by novel compounds; however, genetic-based approaches have not fully revealed the direct cause of inter-individual differences in the sensitivity to cytostatic drugs [3–6]. Therefore, understanding the mechanism of action and being able to predict the impaired effect of commonly administered drugs is crucial for improving patient outcomes.

We turned our attention to cellular metabolism which represents a limiting process in cell proliferation and survival. Recent data have indicated that a number of cytostatic drugs trigger metabolic reprogramming, which impairs their effects and, in the long run, could cause resistance. Our group previously described that leukemia cells treated with L-asparaginase (ASNase) reprogrammed their metabolism and increased fatty acid oxidation (FAO) together with autophagy in order to compensate for asparagine and glutamine depletion [7]. Pharmacological inhibition of FAO increased the sensitivity to ASNase in leukemia cells, which supported its pro-survival effect and its potential role in the mechanism of resistance. A similar phenomenon was described in chronic lymphoblastic leukemia, where the administration of dexamethasone led to the increase in FAO [8]. Since treatment can dramatically influence the metabolic setup, we assume that reciprocally, the metabolic predisposition of cancer cells could also interfere with their response to treatment.

Our aim was to investigate how the basal metabolic profile of leukemic cells interferes with the effectiveness of the compounds currently used for treatment. We focused on cytostatic drugs used in the treatment of childhood leukemia with an emphasis on ASNase. ASNase represents a crucial drug used in the treatment of ALL; it is also incorporated in the front-line treatment of adult leukemia [9, 10]. Unfortunately, the sensitivity to ASNase differs significantly among ALL patients as described by Ramarkers et al. [11]. A specific phenotypic subgroup of ALL derived from a T lymphoid lineage (T-ALL) was also shown to be more resistant to ASNase treatment [12, 13]. In the current study, we have determined the metabolic phenotype of different types of leukemia in order to better understand their differences, metabolic demands, and their connection with the selective sensitivity of ASNase.

## Methods

### Cell culture

Human B-cell precursor leukemia cell lines (BCP-ALL; TOM-1, HB11;19, RS4;11, UOC-B6, REH, SUP-B15, NALM6), T-cell leukemia cell lines (HPB-ALL, CCRF-CEM, JURKAT, MOLT-4), AML cell lines (MV4;11, KASUMI-1, NB-4, THP-1, MOLM-13) and cell lines derived from the blast crisis of chronic myeloid leukemia (CML), which manifest as AML (K-562, LAMA-84) and as ALL (BV-173), were used (Table S1). The HB11;19 cell line was kindly provided by Dr. Anthony Ford from the Institute of Cancer Research (London, UK), and the UOC-B6 cell line was provided by Dr. Ondrej Krejci (Massachusetts General Hospital, Boston) [14]. The rest of the cell lines were purchased from the German Collection of Microorganisms and Cell Cultures (DSMZ, Braunschweig, Germany). The cell lines were negative for mycoplasma contamination and cultivated in RPMI-1640 medium with GlutaMAX™ supplemented with 10% fetal calf serum, penicillin (100 U/mL) and streptomycin (100 μg/mL) under controlled conditions (37 °C, 5% CO_2_). The cultured cells were split every 2 to 3 days and maintained in exponential growth phase.

### Patient samples

Bone marrow or peripheral blood samples from untreated children initially diagnosed with BCP-ALL, T-ALL or AML were collected from the Czech Pediatric Hematology Centers. The inclusion criteria were the percentage of blasts higher than 80% and high cellularity. Within 24 h after aspiration, without freezing, the mononuclear cells were isolated by density gradient centrifugation using Ficoll-Paque PLUS (GE Healthcare, UK). All samples were obtained with the informed consent of the children’s parents or guardians. The study no. 201528848A was approved by the Ethical Committee of the University Hospital Motol, Prague, Czech Republic. Healthy controls were isolated from buffy coats (mixture of healthy individuals) using Ficoll-Paque PLUS (GE Healthcare, UK). To enrich samples for B-lymphocytes, buffy coat was pre-treated with RosetteSep™ Human B Cell Enrichment Cocktail (StemCell Technologies, USA) prior to Ficoll-Paque PLUS.

The isolated blasts were maintained in RPMI-1640 medium with GlutaMAX™ supplemented with 10% fetal calf serum, penicillin (100 U/mL) and streptomycin (100 μg/mL). For the MTS assay, insulin-transferrin-sodium selenite supplement was added to the culture media (Sigma-Aldrich, St Louis, MO, USA).

### Mitochondrial FAO measurement

The cells were incubated for 4 h in culture medium containing 100 μM palmitic acid, 1 mM carnitine and 1.7 μCi [9,10(n)-^3^H] palmitic acid (GE Healthcare, UK) in the presence or absence of etomoxir (100 μM, Sigma-Aldrich, MO, USA). Medium was collected to analyze the amount of released ^3^H_2_O that was formed during the cellular oxidation of [^3^H]-palmitate [15, 16]. The procedure was performed as described previously [7]. The measurement was performed in 3 independent experiments.

### TMRE staining

The cells were incubated for 30 min in culture medium with or without 1 μM tetramethylrhodamine ethyl ester (TMRE; ThermoFisher Scientific Inc., MA, USA) and were then washed with PBS and analyzed on a flow cytometer according to the manufacturer’s instructions. The level of TMRE staining was expressed as the mean of the TMRE signal in the live cells. The measurement was performed in 3 independent experiments.

### Cell survival and proliferation

To evaluate the cytotoxicity of ASNase, vincristine (VCR), and daunorubicin (DNR), MTS (dimethylthiazol carboxymethoxyphenyl sulfophenyl tetrazolium) assays were performed using a CellTiter 96 AQueous One Solution Cell Proliferation Assay (Promega Corporation, Wisconsin, USA) according to the manufacturer’s instructions and our previous publication [7]. Range of ASNase concentration used in the MTS assay was 0 - 4 IU/ml (5-fold dilutions), for VCR it was 0 - 50 nM (5-fold dilutions) and 0 - 3 μM (5-fold dilutions) for DNR. We seeded 1.2 × 10^4^ cells of leukemia cell lines and 1 × 10^5^-3 × 10^5^ patient cells. To evaluate the combined cytotoxicity of the 2 drugs, the number of live cells was determined by flow cytometry using DAPI (ThermoFisher Scientific Inc., MA, USA) and AccuCount Blank Particles (Spherotech Inc., IL, USA). The MTS assay was performed in at least 3 independent experiments. Cell counts were done in biological triplicates.

### Seahorse extracellular flux analysis

Glycolytic and mitochondrial respiration parameters of the leukemia cell lines were measured on a Seahorse Analyzers XFe24 and XFp (Agilent Technologies, Inc., CA, USA) using a Glycolysis stress test and a Cell mito stress test. For the Glycolysis stress test, cells were seeded in XF Base medium, pH 7.4, and for the Cell mito stress test, cells were seeded in XF Assay medium, pH 7.4, supplemented with 10 mM glucose, 1 mM HEPES, pH 7.4, 1 mM pyruvate and 0.1% BSA. The cells were plated at a density of 300,000 cells/well in XFe24 or 40,000 cells/well in XFp tissue culture plates coated with CellTak (Corning GmbH, Wiesbaden, GER), according to the Agilent Seahorse protocol for seeding suspension cells.

The glycolytic and mitochondrial respiration parameters of the primary leukemia cells were measured on the Seahorse Analyzer XFp using the same tests and media as in the case of the cell lines. The primary cells were plated at a density of 500,000 cells/well in XFp tissue culture plates. The procedure was performed as described previously [17].

Final concentrations of the injected drugs were 10 mM glucose, 1 μM (for cell lines) or 2 μM (for primary cells) Oligomycin A and 100 mM 2-deoxy glucose (2-DG) in the Glycolysis stress test and 2 μM Oligomycin A, 1–4.5 μM FCCP (depending on the cell line) and 1 μM Rotenone combined with 1 μg/ml Antimycin A in the Cell mito stress test. All cell lines were measured at least in biological triplicates and 5 technical replicates on the Seahorse Analyzer.

### Genomic DNA isolation and mtDNA quantification

Genomic DNA was isolated from leukemia cell lines using the QIAamp DNA Mini Kit (Qiagen GmbH, Germany) according to the manufacturer’s instructions. To quantify the mtDNA content, 2 genes were used as mitochondrial targets (*16S rRNA* and *D-loop* genes) and the *GAPDH* gene served as a nuclear target. Quantification was performed using real-time PCR as described elsewhere [18].

### Electrophoresis and western blotting

Protein lysates were prepared as previously described [19]. The proteins (30 μg per well) were resolved by NuPAGE Novex 4–12% Bis-Tris Gels (ThermoFisher Scientific Inc., MA, USA) and transferred to a nitrocellulose membrane (Bio-Rad, CA, USA). The membrane was probed overnight with the primary antibodies listed in Table S2. The bound antibodies were detected with the appropriate secondary antibodies conjugated to horseradish peroxidase (Bio-Rad, CA, USA) and visualized using an enhanced chemiluminescence reagent and documented by Uvitec (Cambridge, UK).

### Statistical analysis

Hierarchical clusters were generated in R using the Pheatmap package (distance measure: “Euclidean”, clustering method: “ward.D2”). Linearization method was used to calculate adjusted (bonferroni) p-values for Oligomycin A effect to ASNase, VCR and DNR sensitivity of leukemia cells (Fig. 3). Spearman rank correlations were calculated in R using the method “spearman”. P-values in Figure S1K were calculated using an unpaired, two-tailed, Mann-Whitney test in GraphPad Prism 6. Canonical Correlation analysis was done in R.

## Results

### Characterization of the basal metabolic state of leukemia cell lines

We used 19 human leukemia cell lines of different origin (8 BCP-ALL (ALL derived from B-lymphocytes; 1 BCP-ALL originated from a CML blast crisis), 4 T-ALL and 7 of myeloid origin (5 AML; 2 CML blast crisis manifesting as AML)); in all of them, we determined glycolytic function, mitochondrial respiration and FAO (Figure S1). Glycolytic and mitochondrial functions were measured on a Seahorse Analyzer XFe24 using Glycolysis stress test and Cell Mito stress test, respectively. This allowed us to measure and calculate different parameters within the glycolytic (basal acidification, glycolysis, glycolytic capacity, glycolytic reserve) and mitochondrial (basal respiration, ATP-linked respiration, maximal respiration, spare capacity) functions. FAO activity was measured as the oxidation of [^3^H]-palmitate.

**Fig. 1:**
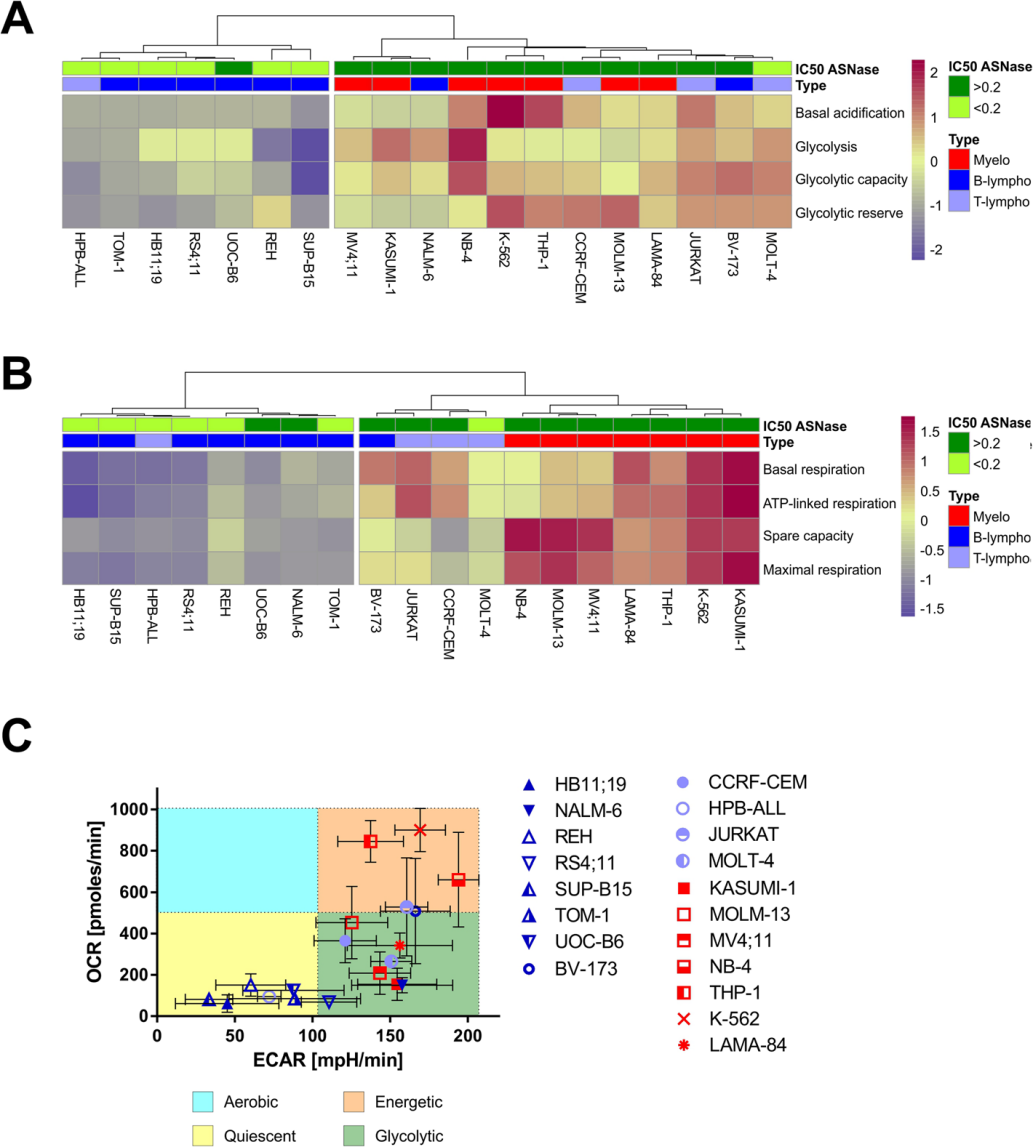
Basal metabolic state of leukemia cell lines. **a** Hierarchical cluster analysis of leukemia cell lines based on the parameters calculated from their glycolytic function. **b** Hierarchical cluster analysis of leukemia cell lines based on the parameters calculated from their mitochondrial function. Type of leukemia and IC50 ASNase [IU/ml] are indicated for each cell line. **c** Oxygen consumption rate (OCR) level against the extracellular acidification rate (ECAR) level of the leukemia cell lines after glucose injection in the measurement of Glycolysis Stress Test. The type of leukemia is indicated in (**a**) and (**b**). All the Seahorse measurements were done at least in biological triplicates and 5 technical replicates and are presented as a mean ± SD

Hierarchical clustering analysis (HCA) revealed that glycolysis and mitochondrial respiration, but not FAO, clustered leukemia cells separately according to the hematopoietic lineage from which they were derived (Fig. 1a, b, S1). Myeloid leukemia and T-ALL cell lines had overall higher values of glycolytic and cell respiration parameters and clustered together, while B-ALL cell lines with generally lower values gathered into the second cluster. Nevertheless, there were two exceptions. The HPB-ALL, a T-ALL cell line which clustered with the B-ALL and the NALM-6 and BV-173 B-ALL cell lines, which clustered together with the myeloid leukemia and T-ALL cell lines. In the case of NALM-6, this result was only achieved according to glycolytic function. Next, the OCR (oxygen consumption rate)/ECAR (extracellular acidification rate) ratio was calculated from the OCR and ECAR values, which were determined during the measurement of the glycolytic function after glucose addition (Figure S1). We plotted OCR against ECAR dividing leukemia cell lines according to their energy status. Cells preferentially oxidizing glucose in mitochondria have an aerobic status, while those utilizing glucose for fermentation to lactate have a glycolytic status. When both OCR and ECAR values are low, the cells are quiescent, whereas if both values are high, the cells are energetic, meaning they are very actively using both glycolysis and oxidative phosphorylation (OXPHOS, Fig. 1c).

Next, we tested which cellular processes could participate in a given metabolic state. The relative activity of major signalling pathways influencing basal metabolism was determined in 15 leukemia cell lines of different origin (6 B-ALL, 4 T-ALL, 5 AML; Figure S1K). The western blots were quantified by densitometry (normalized to β-Actin level). Importantly, we found higher levels of constitutively phosphorylated AKT (p-AKT) in all of the T-ALL cell lines when compared to the B-ALL cell lines (p = 0.0095). Furthermore, the levels of phosphorylated GSK-3β (glycogen synthase kinase 3 beta; p-GSK-3β) at Ser9 in the B-ALL cell lines were significantly lower compared to the levels in the AML cell lines (p = 0.0455) Activation of GSK-3β by dephosphorylation inhibits glycogen synthesis. We also detected elevated phospho-S6 (protein S6; p-S6), a downstream target of the PI3K/AKT/mTOR pathway, in the AML cell lines compared to the B-ALL cell lines (p = 0.0303). Moreover, we found significant difference in the levels of c-MYC (an activator of glycolysis) between the B-ALL and the T-ALL cell lines (p = 0.0190). We did not see any significant difference in the levels of p-AMPK (an AMP-activated protein kinase with a role in cellular energy homeostasis) among leukemia types.

### Association of the metabolic profile of leukemia cells and their sensitivity to cytostatic drugs

**Fig. 2:**
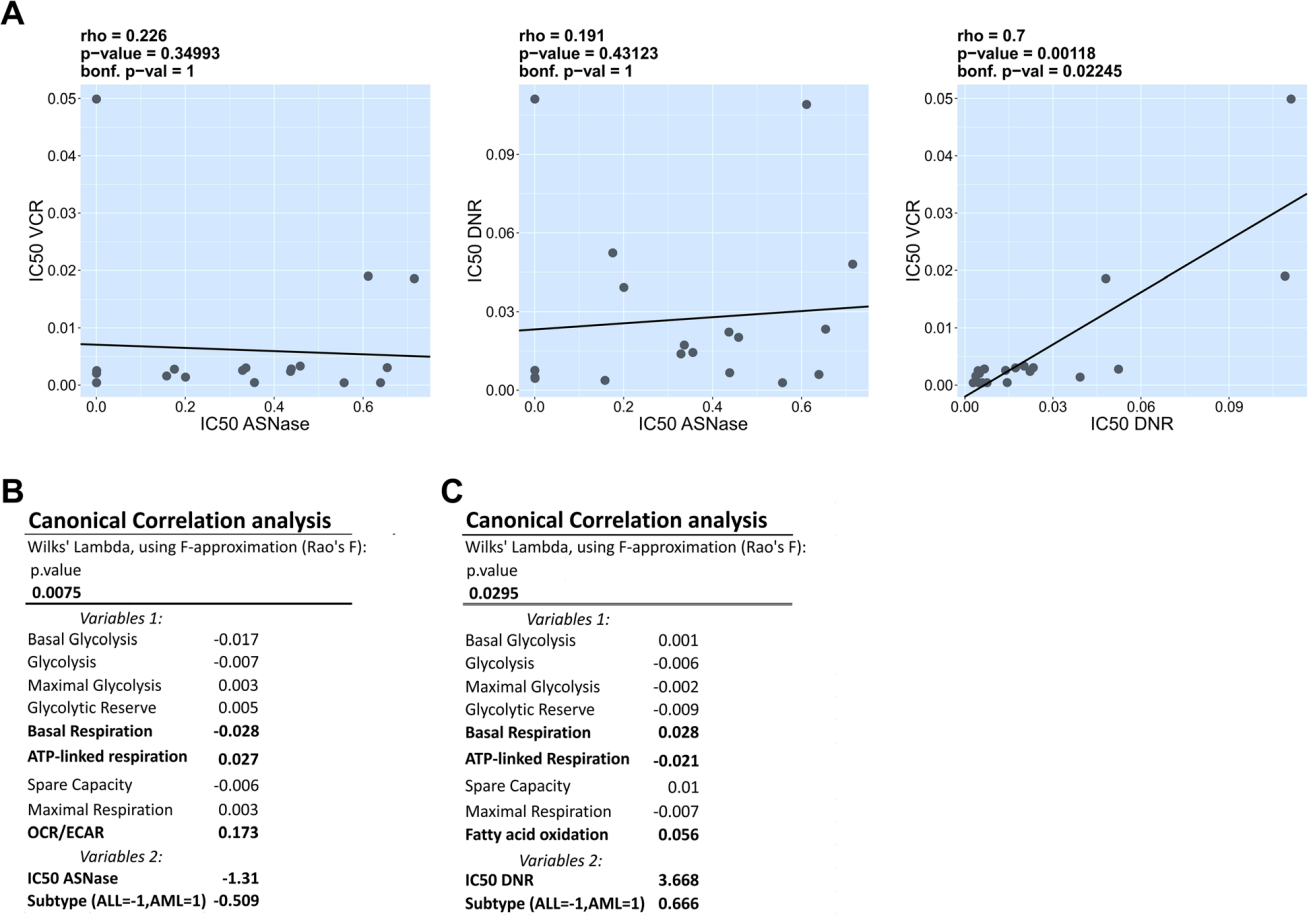
Association between the metabolic profile of the leukemia cells and the sensitivity to cytostatic drugs. **a** Spearman rank correlation calculations of leukemia cell lines IC50 of ASNase [IU/ml] with VCR [μM], ASNase [IU/ml] with DNR [μM], VCR [μM] with DNR [μM], followed by a Bonferroni multiple comparison test. **b**, **c** Canonical correlation analysis: we specified all the metabolic parameters as the first set of variables and IC50 of ASNase (or IC50 of DNR) plus leukemia cell types as the second set. Variables 1, which are mostly correlated with variables 2, are highlighted. Canonical coefficients are displayed across all sets of variables. Their values can differ due to different types of parameters. Negative or positive coefficients express negative or positive correlations with parameters from the other set (**b**) The strongest influence from variable set 1 were OCR/ECAR, basal respiration and ATP-linked respiration and from variable set 2 were IC50 and cell type. **c** The strongest influences from variable set 1 were FAO, basal respiration and ATP-linked respiration and from variable set 2 were IC50 and cell type

Next, we investigated if there is a relationship between the metabolic phenotype of leukemic cells and the treatment sensitivity. First, we measured the sensitivity of all 19 leukemia cell lines to ASNase, Vincristine (VCR) and Daunorubicin (DNR). The last 2 drugs are included in the treatment of a broader spectrum of cancers. First, sensitivity to ASNase did not correlate with sensitivity to VCR and DNR, which demonstrated that the tested leukemia cell lines are not generally sensitive or resistant to cytostatic drugs. However, due to their intrinsic properties they respond independently to drugs with different mechanisms of action (Table 1a; Fig. 2a). We observed that myeloid cell lines are least sensitive to ASNase, followed by T-ALL cells, with B-ALL cell lines being the most sensitive ones (Table 1b).

**Table 1:** Sensitivity of leukemia cell lines to cytostatic drugs

A					B						
Cell line	Type	IC50 ASNase [U/ml]	IC50 VCR [μM]	IC50 DNR [μM]	Type	Median			Mean ± SEM		
HPB-ALL	T-ALL	0.000192	0.049900	0.111165		IC50 ASNase [IU/ml]	IC50 VCR [μM]	IC50 DNR [μM]	IC50 ASNase [IU/ml]	IC50 VCR [μM]	IC50 DNR [μM]
REH	B-ALL	0.000231	0.000420	0.007571	B-ALL	0.000677	0.001601	0.004953	0.1206 ± 0.0613	0.001437 ± 0.000376	0.007675 ± 0.001731
RS4;11	B-ALL	0.000378	0.002049	0.004953	T-ALL	0.3061	0.003056	0.03728	0.2677 ± 0.1099	0.014600 ± 0.011770	0.051480 ± 0.021210
SUP-B15	B-ALL	0.000549	0.000428	0.004655	AML	0.4385	0.001399	0.006661	0.4344 ± 0.0779	0.001615 ± 0.000562	0.014400 ± 0.006673
HB11;19	B-ALL	0.000677	0.002534	0.004509							
TOM-1	B-ALL	0.158103	0.001601	0.003750							
MOLT-4	T-ALL	0.175502	0.002784	0.052362							
THP-1	AML	0.200475	0.001399	0.039269							
NALM-6	B-ALL	0.328894	0.002578	0.013874							
MV4–11	AML	0.336387	0.003011	0.017245							
UOC-B6	B-ALL	0.355603	0.000450	0.014414							
JURKAT	T-ALL	0.436766	0.002393	0.022205							
KASUMI-1	AML	0.438479	0.002823	0.006661							
CCRF-CEM	T-ALL	0.458528	0.003328	0.020190							
MOLM-13	AML	0.557342	0.000418	0.002844							
LAMA-84	CML in blast crisis (manifests as ALL)	0.611470	0.019001	0.109103							
NB4	AML	0.639279	0.000424	0.005995							
BV-173	CML in blast crisis (manifests as ALL)	0.654297	0.003051	0.023315							
K-562	CML in blast crisis (manifests as AML)	0.715221	0.018563	0.048036

Next we asked if there was any relationship between the overall metabolic state of the leukemia cells (including all parameters measured) and their sensitivity to cytostatic drugs. For this purpose, we used Canonical Correlation analysis (CCA), which explores the relationship between 2 multivariate sets of variables. In our case, set 1 included all metabolic parameters, and set 2 consisted of the lineage origin and IC50s (Fig. 2b, c). Higher sensitivity to ASNase, preferentially presented in lymphoid leukemias, correlated most significantly with lower basal respiration, higher ATP-linked respiration and with a preference for oxidative (OCR/ECAR) over glycolytic metabolism (p = 0.0075, Fig. 2b). Higher sensitivity to DNR represented in AML correlated with lower basal respiration, higher ATP-linked respiration and lower FAO (p = 0.0295; Fig. 2c). Sensitivity to VCR did not correlate with any metabolic activity.

### Functional study of the correlation between ATP synthase activity and the sensitivity to ASNase

For a functional confirmation of the results from CCA, we chose to inhibit ATP synthase activity in order to test the relationship between ATP-linked respiration and sensitivity to ASNase. First, we determined the optimal concentration of Oligomycin A (a specific inhibitor of ATP synthase) that effectively decreased the level of ATP, but at the same time did not completely inhibit cell proliferation. Thus, we counted live cells after 48 and 72 h of treatment with different concentrations of Oligomycin A (20 nM–2 μM). The results showed that 20 nM Oligomycin A inhibited the proliferation of leukemia cells to the same level as higher concentrations (Figure S2A). When measuring mitochondrial respiration, cells treated with 20 nM Oligomycin A had lower basal respiration than untreated cells. Moreover, this lowered respiration was not further reduced after 2 μM Oligomycin A injection, which means that ATP synthase was already inhibited (Figure S2A). OCR of untreated cells dropped dramatically after 2 μM Oligomycin A injection, reaching the level of 20 nM Oligomycin A-treated cells. Based on these data, we selected 20 nM Oligomycin A for further experiments.

**Fig. 3:**
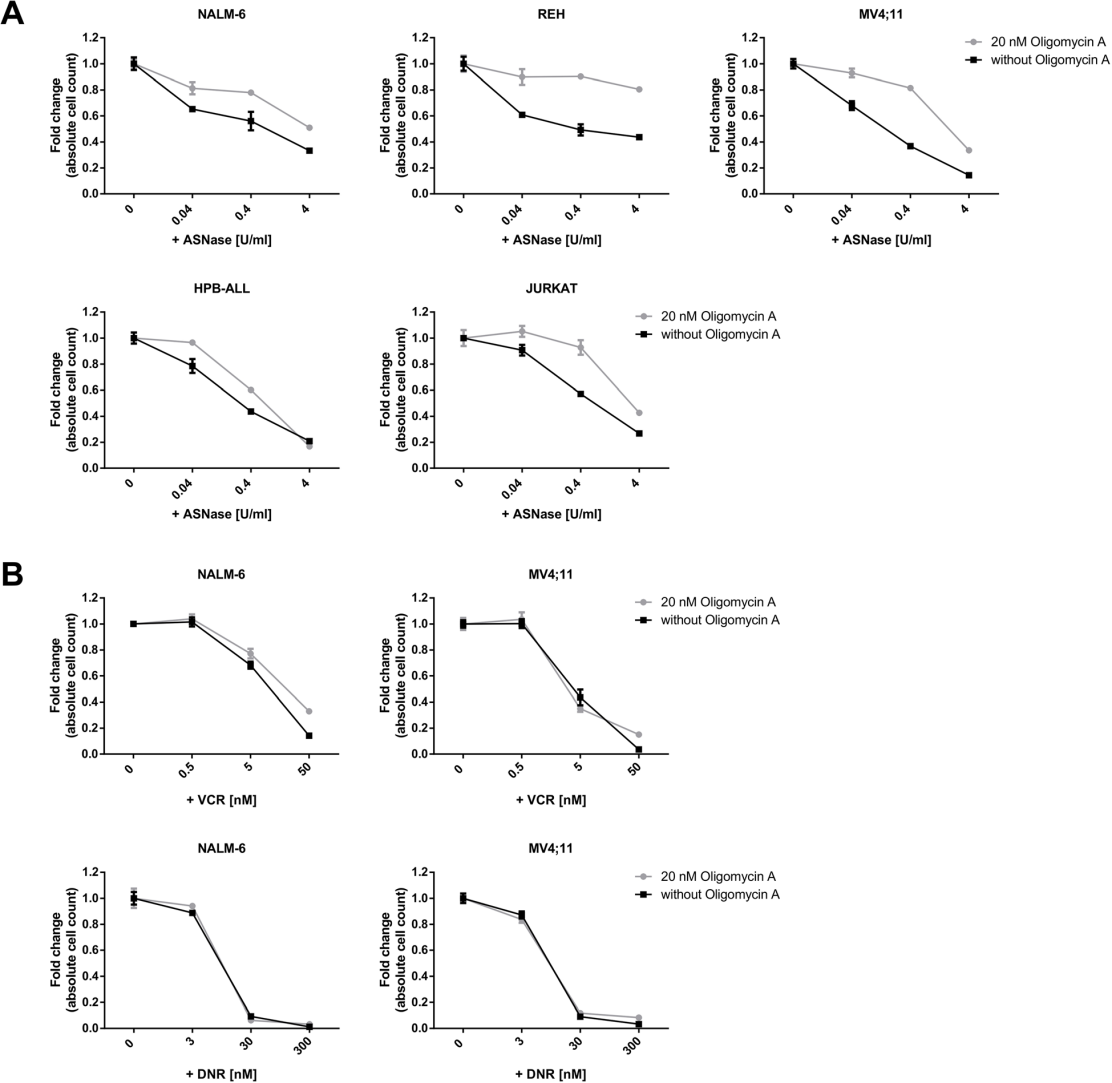
Functional study on the correlation between ATP synthase activity and sensitivity to ASNase. **a** Effect of Oligomycin A (20 nM) on the sensitivity of leukemia cell lines (REH, NALM-6, MV4;11, JURKAT and HPB-ALL) to ASNase. **b** Effect of Oligomycin A (20 nM) on the sensitivity of leukemia cell lines (NALM-6, MV4;11) to VCR and DNR. Cells were pre-treated with Oligomycin A for 1 h or left untreated and then co-treated with ASNase (or VCR, DNR) for the indicated time (48 h; JURKAT and HPB-ALL for 72 h). Absolute cell counts were obtained from 3 independent experiments; data were normalized to untreated controls and are presented as mean ± SD

Oligomycin A treatment was tested on 5 human leukemia cell lines of different origin (2 B-ALL, 2 T-ALL, 1 AML). Leukemia cells were pre-treated with 20 nM Oligomycin A for 1 h and then co-treated with different concentrations of ASNase or left untreated for 48 or 72 h. Then, live cells were counted using flow cytometry. The results showed that Oligomycin A pre-treatment increased the resistance of leukemia cells to ASNase compared to cells treated with ASNase alone (adjusted (bonferroni) p-value< 10^− 4^; representative graphs of all treated cell lines are shown in Fig. 3a). This result suggests that cells with lower ATP synthase activity have a lower sensitivity to ASNase. The effect was evident in all tested cell lines. However, the cytostatic effect of VCR and DNR was not disturbed by Oligomycin A pre-treatment in the tested leukemia cell lines (Fig. 3b). Surprisingly, pre-treatment with 10 ng/ml of Antimycin A (inhibitor of ETC complex III) did not decrease the sensitivity of leukemia cells to ASNase compared to cells treated with ASNase alone (Figure S3). These results attest that the decreased sensitivity to ASNase in cells pre-treated with Oligomycin A is not due to reduced cell growth, since cells treated with both agents, i.e. Antimycin A (10 ng/ml) and Oligomycin A (20 nM), show similar growth reduction compared to non-treated cells (Figure S2).

### Association of mitochondrial membrane potential with the sensitivity to ASNase

**Fig. 4:**
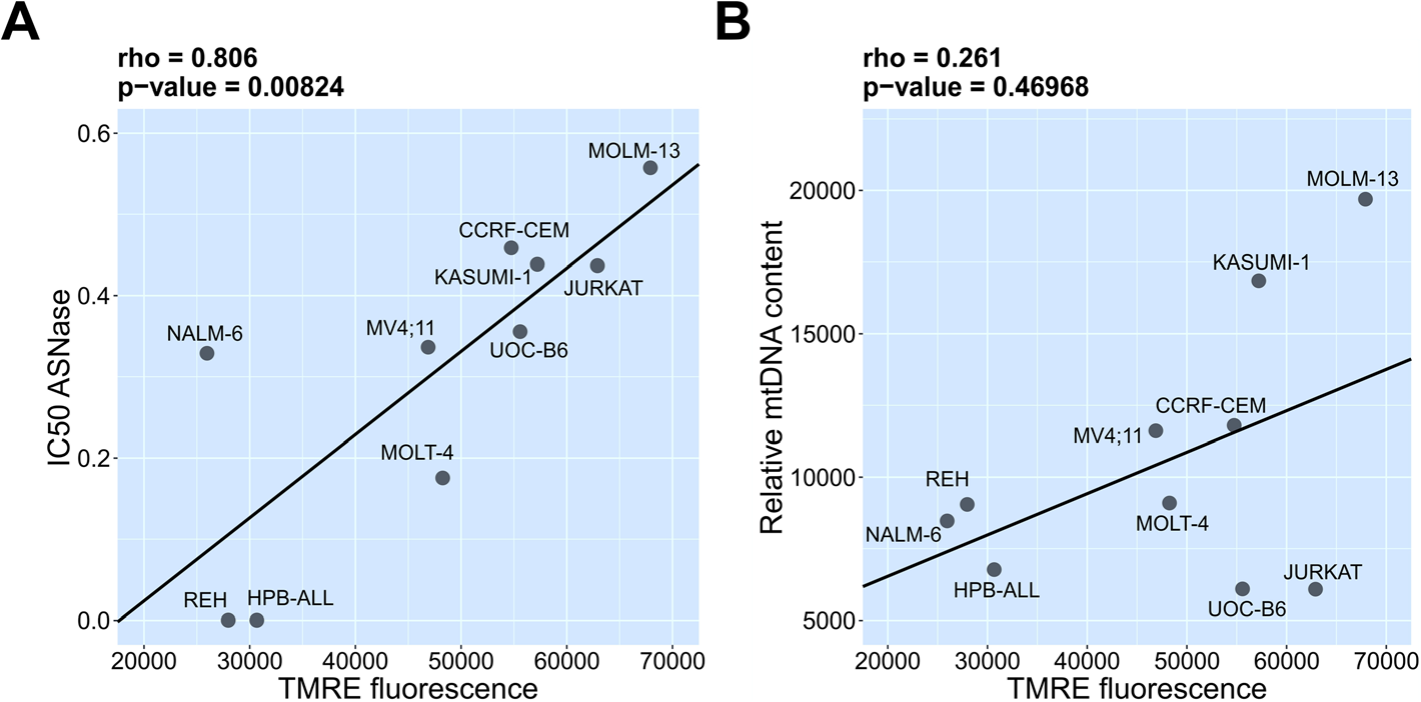
Association of mitochondrial membrane potential with sensitivity to ASNase. Spearman rank correlation calculations of leukemia cell lines (NALM-6, REH, UOC-B6, CCRF-CEM, HPB-ALL, JURKAT, MOLT-4, KASUMI-1, MOLM-13, MV4;11). **a** IC50 ASNase [IU/ml] with TMRE fluorescence. **b** TMRE fluorescence with relative mtDNA content. Measurement was performed in biological triplicates

Basal mitochondrial membrane potential (MMP) was measured as TMRE fluorescence in 10 human leukemia cell lines of different origin (3 B-ALL, 4 T-ALL, 3 AML). Additionally, mitochondrial content was determined as the amount of mtDNA by real-time PCR. TMRE is an MMP-dependent dye and its fluorescence intensity is in proportion to both MMP and mitochondrial mass. TMRE fluorescence alone, or TMRE fluorescence normalized to mtDNA amount, clustered leukemia cell lines regardless of their origin (data not shown). On the other hand, in the tested leukemia cell lines, TMRE fluorescence significantly correlated (rho = 0.806; p-value = 0.00824) with sensitivity to ASNase (Fig. 4a). This means that cell lines with a higher TMRE fluorescence have in general a lower sensitivity to ASNase. Nevertheless, because TMRE fluorescence did not correlate with mtDNA quantity, it can be concluded that the observed TMRE changes are a representation of basal MMP in leukemia cells (Fig. 4b).

### Characterization of the metabolic profile of leukemia patients

**Fig. 5:**
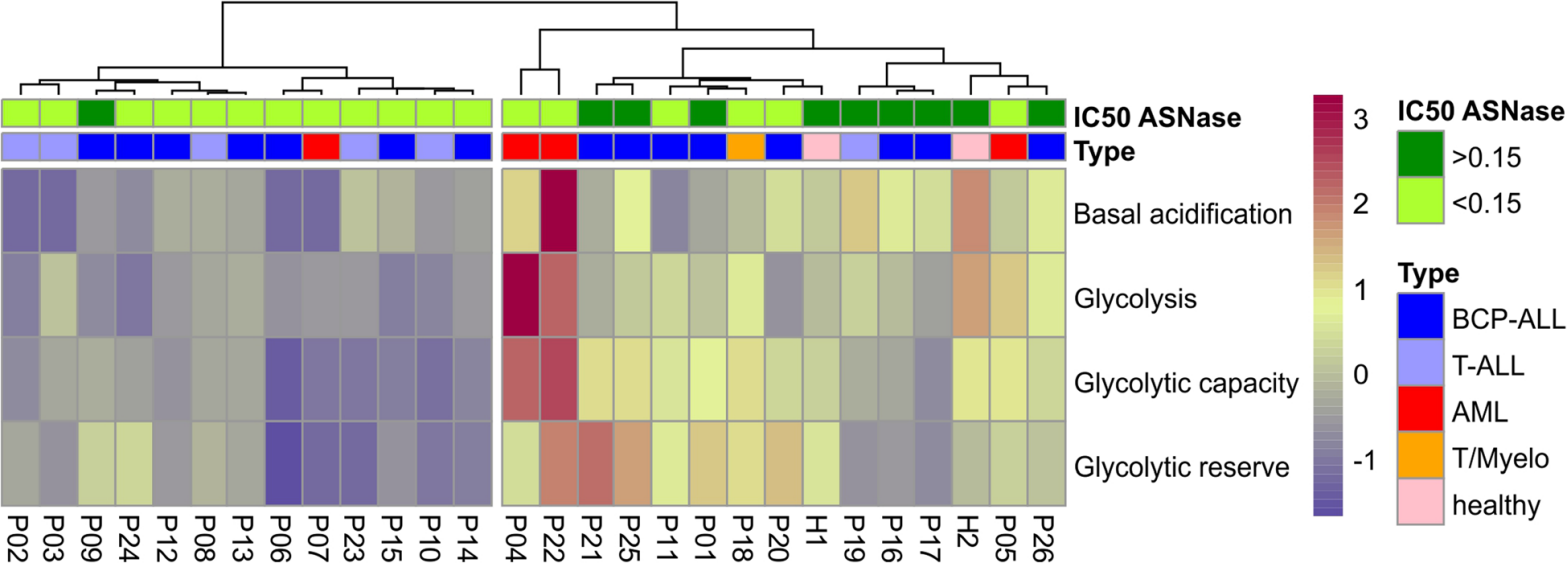
Characterization of the metabolic profile of leukemia patients. Hierarchical cluster analysis of primary leukemia cells and healthy control samples which was based on the parameters of the glycolytic function. Type of the leukemia and IC50 ASNase [IU/ml] are indicated for each patient. For more information, see Table 2

Although measuring the metabolic state of primary leukemia cells is quite challenging, we assessed the glycolytic and mitochondrial function of cells from 26 childhood leukemia patients (14 BCP-ALL, 7 T-ALL, 4 AML, 1 T/Myelo) and 2 healthy controls (B-cells, mononuclear cells)). We also determined the sensitivity of primary leukemia cells to ASNase by MTS assay (Table 2). HCA gathered patient samples according to their metabolic profile into 2 main clusters. The first cluster consisted, almost exclusively, of ALL patients (12/13). The second cluster included AML patients (3/4), a biphenotypic patient (1/1), healthy controls (2/2) and also ALL patients (9/21) (Fig. 5). Interestingly, ALL patients more sensitive to ASNase (11/13) clustered to the low glycolytic cluster, while patients less sensitive to ASNase (7/8) fell into the high glycolytic cluster. Mitochondrial respiration did not divide the ALL patients as did the glycolytic function (Figure S4).

**Table 2:** Characterization of leukemia patients

Patient	Type of leukemia	Genetic aberations	Age	Sex	Percentage of blasts during diagnosis	Peripheral blast count (×10^**9**^/l)	IC50 ASNase [IU/ml]
P01	pre-B ALL	KMT2A/MLLT3	1	F	91%	161.07	0.148802
P02	interT-ALL	none detected	6	F	81%	51.03	0.014910
P03	T-ALL	none detected	20	M	92%	NA	0.000291
P04	AML M4	CBFβ-MYH11	10	F	86%	122.12	0.114985
P05	AML M1	DEK-NUP214. FLT3-ITD	17	M	85%	28.82	0.000255
P06	pro-B ALL	ETV6-RUNX1	11	F	93%	8.18	0.040462
P07	AML M4	NPM1	17	F	83%	20.75	0.037307
P08	interT-ALL	none detected	8	F	94%	68.43	0.003352
P09	pre-B ALL	IKZF1del	1	M	89%	NA	0.284902
P10	interT-ALL	none detected	15	F	91%	NA	0.023467
P11	cALL	none detected	7	M	95%	40.28	0.125948
P12	cALL	ETV6-RUNX1	5	F	95%	4.18	0.117476
P13	cALL	ETV6-RUNX1	3	M	84%	4.20	0.001058
P14	cALL	ETV6-RUNX1	10	M	89%	3.39	0.105594
P15	cALL	ETV6-RUNX1	8	F	91%	56.60	0.000184
P16	cALL	hyperdiploid	16	F	93%	26.55	0.235677
P17	cALL (partial ProB)	none detected	8	F	97%	281.30	> 0.15
P18	T/Myelo	FLT3-ITD	9	M	88%	9.90	0.000242
P19	preT-ALL	none detected	12	F	75%	139.95	> 0.15
P20	cALL	ETV6-RUNX1	4	M	96%	31.97	0.000172
P21	pre-B ALL	none detected	2	M	91%	108.98	0.147981
P22	AML M5	KMT2A/MLLTA10	1	F	83%	366.28	0.000181
P23	interT-ALL	none detected	4	M	81%	93.64	0.104566
P24	cALL	ETV6-RUNX1	8	M	96%	NA	0.005477
P25	cALL	none detected	11	F	95%	6.042	> 0.15
P26	pro-B ALL	KMT2A/AFF1 (MLL-AF4)	< 1	F	92%	404.10	0.388752
H1	Healthy B-cells			NA			Resistant
H2	Healthy mononuclear cells			NA			Resistant

## Discussion

The aim of this study was to elucidate if metabolic predisposition of leukemia cells (both cell lines and primary cells) influences their response to therapy. We wondered whether there is a basal metabolic profile that would predict which leukemia patients are sensitive or resistant to a given treatment. Since our previous work showed that ASNase affects the bioenergetics of leukemic cells, we were particularly interested in the link between the sensitivity to ASNase and the basal metabolic status of the cells as assessed before treatment. In addition to ASNase, we also tested the sensitivity to VCR and DNR, cytostatic drugs with a different mechanism of action, which are used in the treatment of a wide variety of cancers. We first examined the metabolic activity, including glycolysis, mitochondrial respiration and FAO in 19 cell lines. We performed HCA in order to cluster cell lines with a similar metabolic profile. Based on both glycolytic function and mitochondrial respiration, the cell lines were very similarly gathered into clusters. On the other hand, FAO clustered the cell lines differently. The first cluster, consisting of exclusively lymphoid leukemia cell lines, associated the cell lines with a lower glycolytic function and a lower mitochondrial respiration. The second cluster, grouped the cell lines with a higher glycolytic function and a higher mitochondrial respiration and contained all myeloid leukemia cell lines as well as some T-ALL cell lines. Next, aiming at the confirmation of this phenomenon in the primary samples, we assessed glycolytic function and mitochondrial respiration in leukemia cells isolated from ALL and AML patients and performed HCA. The first cluster which was divided according to glycolytic function consisted mostly of samples from ALL patients and the second cluster combined both, AML patients, ALL patients and healthy controls. After subsequent examination of the samples for their susceptibility to cytostatics, we found that ALLs from the high glycolytic cluster were less sensitive to ASNase treatment than their counterparts in the low glycolytic cluster. Moreover, a similar association was observed also in the tested cell lines. T-ALL lines from the high glycolytic cluster (and high respiration) were resistant to ASNase. Interestingly however, some AML patients were sensitive to ASNase to the same extent as sensitive ALL patients. This can be explained by the FAB classification according to which these AML patients fall into the M1, M4 or M5 subgroups. These morphological subgroups were shown to be more sensitive to ASNase treatment by in vitro testing [20]. This could be potentially clinically interesting since the incorporation of ASNase is still discussed in the treatment of AML.

In cell lines, IC50 of ASNase did not correlate with IC50 of VCR or DNR and also, the sensitivity to these cytostatics was not associated with any specific metabolic phenotype demonstrating that their effect was less influenced by the basal metabolic status of leukemia cells.

We have previously described specific signaling pathways that regulate bioenergetic processes after ASNase treatment [7]. We were therefore interested if a distinct metabolic profile of the leukemic cells would be associated with a different prerequisite activity of the signaling pathways. Indeed, we found a profound difference in p-AKT and c-MYC, the regulators of glycolysis [21, 22], between T- and B-ALL cells and decreased level of p-GSK3b in B-ALL cell lines. In the case of the AML cell lines, the proteins S6 and p-S6 were more expressed than in ALL cells.

Since we found an association between the metabolic predisposition and the consecutive response to ASNase treatment, we next asked which metabolic parameter correlates with IC50 of ASNase and therefore with the potential treatment efficacy. Statistical analysis based on CCA revealed that higher ATP-linked respiration, higher OCR/ECAR and lower basal respiration significantly correlated with increased sensitivity to ASNase. To functionally confirm this relationship we used Oligomycin A, a specific ATP synthase inhibitor. After its administration, we treated the cells with ASNase and examined the changes in cell survival and growth. We confirmed that cells with lower ATP synthase activity, i.e. lower ATP-linked respiration, are more resistant to ASNase. The sensitivity to VCR and DNR did not change, which corresponded with the results of the CCA. Reduced ATP synthase activity, in relation to a resistant phenotype, has been described in colorectal carcinoma cells and 5-fluorouracil [23]. Our assumption is that cells with less active TCA cycle followed by reduced ATP-linked respiration are less dependent on the supply of glucose or glutamine. Thus, these cells could better tolerate glutamine depletion and glucose uptake impairment, both of which are cytotoxic effects of ASNase.

Since Oligomycin A treatment leads to increased MMP [24], we examined the so-called basal MMP of cells using TMRE labeling. We have shown that higher MMP significantly correlates with higher resistance to ASNase. Since TMRE fluorescence measuring is a standardized cytometric method, it could be established as an additional diagnostic marker to help characterize the sensitivity of individual patients to ASNase.

Even though ASNase is a crucial component in the treatment of ALL, its precise administration is still being tested in some specific ALL prognostic-risk groups [25]. Moreover, its incorporation into the therapy of other cancer types is still under investigation [26–29]. This is the first study describing the correlation between the sensitivity to ASNase and the metabolic profile of leukemia cells. Our results revealed that myeloid and lymphoid cells cluster together based on their glycolytic activity and mitochondrial respiration. Interestingly, both ALL cell lines and patients display different glycolytic activity which is associated with their sensitivity to ASNase. Further characterization of the metabolic state of the leukemic blasts, at the time of diagnosis, may help to identify patients with lower sensitivity to ASNase, individuals that are therefore more likely to fail the conventional therapy without any other detectable high-risk factors.

## Conclusions

Our study provides novel findings on the role of cellular metabolism in the treatment response of leukemia patients. We have shown that metabolic phenotype in ALL cells can predict response to ASNase treatment in which less sensitive patients display high glycolytic action when compared to more sensitive patients with low glycolytic action. We also found correlation between ATP-linked respiration and basal MMP with the sensitivity to ASNase. These data support the role of cancer metabolism and open new potential ways in the diagnostic and treatment management of resistant leukemia patients.

## Supplementary information

**Additional file 1: Supplementary Table S1**. Genetic characterization of leukemia cell lines.

**Additional file 2: Supplementary Table S2**. List of primary antibodies.

**Additional file 3: Supplementary Figure S1.** Absolute values of metabolic parameters measured in 19 leukemia cell lines. (A–D) Parameters calculated from glycolytic function. (E–H) Parameters calculated from mitochondrial function. (I) OCR/ECAR ratio calculated after glucose injection during the Glycolysis stress test measurement. (J) FAO rate. AML is presented as black graphs, B-ALL as white and T-ALL as gray graphs. (K) Levels of signaling proteins in the leukemia cell lines were measured by immunoblotting. β-actin was used as a loading control. Relative quantification (normalized to β-actin) was calculated in ImageJ. * p < 0.05, ** p < 0.01, *** p < 0.001. The immunoblot is a representative result of three independent experiments.

**Additional file 4: Supplementary Figure S2.** Effect of OXPHOS inhibitors on the growth and mitochondrial respiration of leukemia cell lines. (A) The effect of Oligomycin A (20 nM, 200 nM, 2 μM) on the growth and on the course of mitochondrial respiration of NALM-6 cells. (B) The effect of Antimycin A (10 ng/ml, 100 ng/ml and 1 μg/ml) on the growth and the course of mitochondrial respiration of NALM-6 cells. Cells were counted 48 and 72 h after the treatment. Cell Mito Stress Test was performed after 24 h of treatment. Measurements were done in three biological replicates and the data are presented as mean ± SD.

**Additional file 5: Supplementary Figure S3.** Functional study on the correlation between ETC complex III activity and sensitivity to ASNase. Effect of Antimycin A (10 ng/ml) on the sensitivity of leukemia cell lines (NALM-6, MV4;11) to ASNase. Cells were pretreated with Antimycin A for 1 h or left untreated and then co-treated with ASNase for 48 h. Absolute cell counts were obtained from three independent experiments; data were normalized to untreated controls and are presented as mean ± SD. Measurements were done in three biological replicates and the data are presented as mean ± SD.

**Additional file 6: Supplementary Figure S4.** Cluster analysis of patient samples according mitochondrial respiration. Hierarchical cluster analysis of primary leukemia cells and healthy control samples based on parameters calculated from mitochondrial function. Type of leukemia and IC50 ASNase [IU/ml] are indicated for each patient. For more information, see Table 2.

## Data Availability

The datasets used and/or analyzed during the current study are available from the corresponding author at reasonable request.

## Abbreviations

ALL: acute lymphoblastic leukemia
BCP-ALL: ALL derived from B-lineage
T-ALL: ALL derived from T-lineage
AML: acute myeloid leukemia
CML: chronic myeloid leukemia
ASNase: L-asparaginase
FAO: fatty acid oxidation
VCR: vincristine
DNR: daunorubicin
OCR: oxygen consumption rate
ECAR: extracellular acidification rate
MMP: mitochondrial membrane potential
