# Supplementary material for: Metabolic profile of leukemia cells influences treatment efficacy of L-asparaginase
Source: BMC Cancer. 2020 Jun 5;20:526. doi: 10.1186/s12885-020-07020-y (PMC7275298; doi:10.1186/s12885-020-07020-y)
Supplement: Supplementary file 1 — Additional file 1: Supplementary Table S1. Genetic characterization of leukemia cell lines. [file 12885_2020_7020_MOESM1_ESM.pdf]

Supplementary Table 1 Genetic characterization of leukemia cell lines

Cell line	Cytogenetics	Molecular genetics
HPB-ALL	pseudodiploid karyotype with 8% polyploidy - 46<2n>XY, der(1)t(1;16)(q22;p11-12)add(16)(p13), del(2)(p24), del(3)(p11), der(5)t(1;5)(q22-24;q32-33), r(16)(?p12?q12)	expression of TLX3
REH	pseudodiploid karyotype - 46(44-47)<2n>X, -X, +16, del(3)(p22), t(4;12;21;16)(q32;p13;q22;q24.3)-inv(12)(p13q22), t(5;12)(q31-q32;p12), der(16)t(16;21)(q24.3;q22) - sideline with inv(5)der(5)(p15q31),+18 - carries t(12;21) and del(12) producing respective ETV6-RUNX1 (TEL-AML1) fusion and deletion of residual ETC6 (TEL)	expression of fusion gene ETV6-RUNX1 (TEL-AML1)
RS4;11	hyperdiploid karyotype - 47/48<2n>X/XX, +8, +18, t(4;11)(q21;q23), i(7q) - sideline without +8/18 - carries KMT2A-AFF1 (MLL-AFF1; MLL-AF4) fusion gene	expression of fusion gene KMT2A-AFF1 (MLL-AFF1; MLL-AF4)
SUP-B15	pseudodiploid karyotype - 46<2n>XY, der(1)t(1;1)(p11;q31), add(3)(q2?7), der(4)t(1;4)(p11;q35), t(9;22)(q34;q11), add(10)(q25), ?del(14)(q23q31), der(16)t(9;16)(q11;p13)	expression of fusion gene BCR-ABL1
HB11;19	not determined	expression of fusion gene MLL-ENL
TOM-1	hyperdiploid karyotype with 7% polyploidy - 47(47-48)<2n>X, -X, +8, +16, del(7)(p14), der(9)del(9)(q13q34)t(9;22)(q34;q11), der(22)t(9;22)(q34;q11) - carries t(9;22) effecting BCR-ABL1 rearrangement	expression of fusion gene BCR-ABL
MOLT-4	flat-moded hypertetraploid karyotype - 89-99<4n>XXYY, +4, +7, +8, +20, +20, del(6)(q16)x2, der(7)t(7;7)(p15;q11)x2	
THP-1	near-tetraploid karyotype - 94(88-96)<4n>XY/XXY, -Y, +1, +3, +6, +6, -8, -13, -19, -22, -22, +2mar, add(1)(p11), del(1)(q42.2), i(2q), del(6)(p21)x2-4, i(7p), der(9)t(9;11)(p22;q23)i(9)(p10)x2, der(11)t(9;11)(p22;q23)x2, add(12)(q24)x1-2, der(13)t(8;13)(p11;p12), add(?18)(q21) - carries t(9;11) associated with AML M5	expression of fusion gene KMT2A-MLLT3 (MLL-MLLT3; MLL-AF9)
NALM-6	near diploid karyotype - 46(43-47)<2n>XY, t(5;12)(q33.2;p13.2)	expression of fusion gene ETV6/PDGFRB
MV4-11	hyperdiploid karyotype - 48(46-48)<2n>XY, +8, +18, +19, -21, t(4;11)(q21;q23)	expression of fusion gene KMT2A-AFF1 (MLL-AFF1; MLL-AF4)
UOC-B6	not determined	expression of fusion gene ETV6-RUNX1 (TEL-AML1)
JURKAT	flat-moded hypotetraploid karyotype with 7.8% polyploidy - 87(78-91)<4n>XX, -Y, -Y, -5, -16, -17, -22, add(2)(p21)/del(2)(p23)x2 - sideline with additional der(5)t(5;10)(q11;p15), del(9)(p11)	
KASUMI-1	hypodiploid karyotype - 45<2n>X, -Y, -9, -13, -16, +3mar, t(8;21)(q22;q22), der(9)t(9;?)(p22;?), der(15)t(?9;15)((?q11;?p11) - carries both partners of 8;21 translocation associated with AML	expression of fusion gene RUNX1-RUNX1T1 (AML1-ETO)
CCRF-CEM	near-tetraploid karyotype with extensive subclonal variation - 90(88-101)<4n>XX, -X, -X, +20, +20, t(8;9)(p11;p24)x2, der(9)del(9)(p21-22)del(9)(q11q13-21)x2 - sideline with +5, +21, add(13)(q3?3), del(16)(q12)	expression of NKX2-5
MOLM-13	hyperdiploid karyotype with 4% polyploidy - 51(48-52)<2n>XY, +8, +8, +8, +13, del(8)(p1?p2?), ins(11;9)(q23;p22p23) - sideline with idem, +19 - carries occult insertion effecting KMT2A-MLLT3 (MLL-MLLT3; MLL-AF9) fusion	FLT3-ITD
LAMA-84	hypertriploid karyotype with 3.6% polyploidy - 73/74(69-77)<3n>XX, -X, +1, -2, +5, +6, +8, +13, -14, +17, +17, -18, +22, +mar, del(7)(p15), der(9)t(9;22)(q34;q11)x2, i(11q), add(13)(q33), del(17)(p12), der(22)t(9;22)(q34;q11)x4 - carries Ph (4 copies)	expression of fusion gene BCR-ABL1
NB4	hypertriploid karyotype with 3% polyploidy - 78(71-81)<3n>XX, -X, +2, +6, +7, +7, +11, +12, +13, +14, +17, -19, +20, +4mar, der(8)t(8;?)(q24;?), der(11)t(11;?)(?->::11p15->11q22.1::11q13->22.1:), der(12)t(12;?)(p11;?), 14p+, t(15;17)(q22;q11-12.1), der(19)t(10;19)(q21.1;p13.3)x2	expression of fusion gene PML-RARA (L-form)
BV-173	hyperdiploid karyotype - 47(46-48)<2n>X/XY, -9, +22, +mar, add(1)(q42), add(8)(p23), t(9;22)(q34;q11), der(22)t(9;22)(q34;q11), der(?)t(9;?)(?p11;?)	expression of fusion gene BCR-ABL1
K-562	hypotriploid karyotype without sharp mode - 61-68<3n>XX, -X, -3, +7, -13, -18, +3mar, del(9)(p11/13), der(14)t(14;?)(p11;?), der(17)t(17;?)(p11/13;?), der(?18)t(15;?18)(q21;?q12), del(X)(p22)	expression of fusion gene BCR-ABL1
