# Supplementary material for: Metabolic profile of leukemia cells influences treatment efficacy of L-asparaginase
Source: BMC Cancer. 2020 Jun 5;20:526. doi: 10.1186/s12885-020-07020-y (PMC7275298; doi:10.1186/s12885-020-07020-y)
Supplement: Supplementary file 2 — Additional file 2: Supplementary Table S2. List of primary antibodies. [file 12885_2020_7020_MOESM2_ESM.pdf]

Primary antibody	Supplier	Description	Dilution
Akt (pan) (40D4)	Cell Signaling Technology	Mouse monoclonal Ab	1:1000
Phospho-Akt (Ser473) (D9E) XP®	Cell Signaling Technology	Rabbit monoclonal Ab	1:1000
S6 Ribosomal Protein (5G10)	Cell Signaling Technology	Rabbit monoclonal Ab	1:1000
Phospho-S6 Ribosomal Protein (Ser235/236) (D57.2.2E) XP®	Cell Signaling Technology	Rabbit monoclonal Ab	1:1000
c-Myc Antibody	Cell Signaling Technology	Rabbit polyclonal Ab	1:1000
AMPKα (23A3)	Cell Signaling Technology	Rabbit monoclonal Ab	1:1000
Phospho-AMPKα (Thr172) (40H9)	Cell Signaling Technology	Rabbit monoclonal Ab	1:1000
GSK-3β (27C10)	Cell Signaling Technology	Rabbit monoclonal Ab	1:1000
Phospho-GSK-3β (Ser9) (5B3)	Cell Signaling Technology	Rabbit monoclonal Ab	1:1000
β-Actin (clone AC-74, ascites fluid)	Sigma-Aldrich	Mouse monoclonal Ab	1:5000
