# Supplementary figures and images for: Metabolic profile of leukemia cells influences treatment efficacy of L-asparaginase
Source: BMC Cancer. 2020 Jun 5;20:526. doi: 10.1186/s12885-020-07020-y (PMC7275298; doi:10.1186/s12885-020-07020-y)

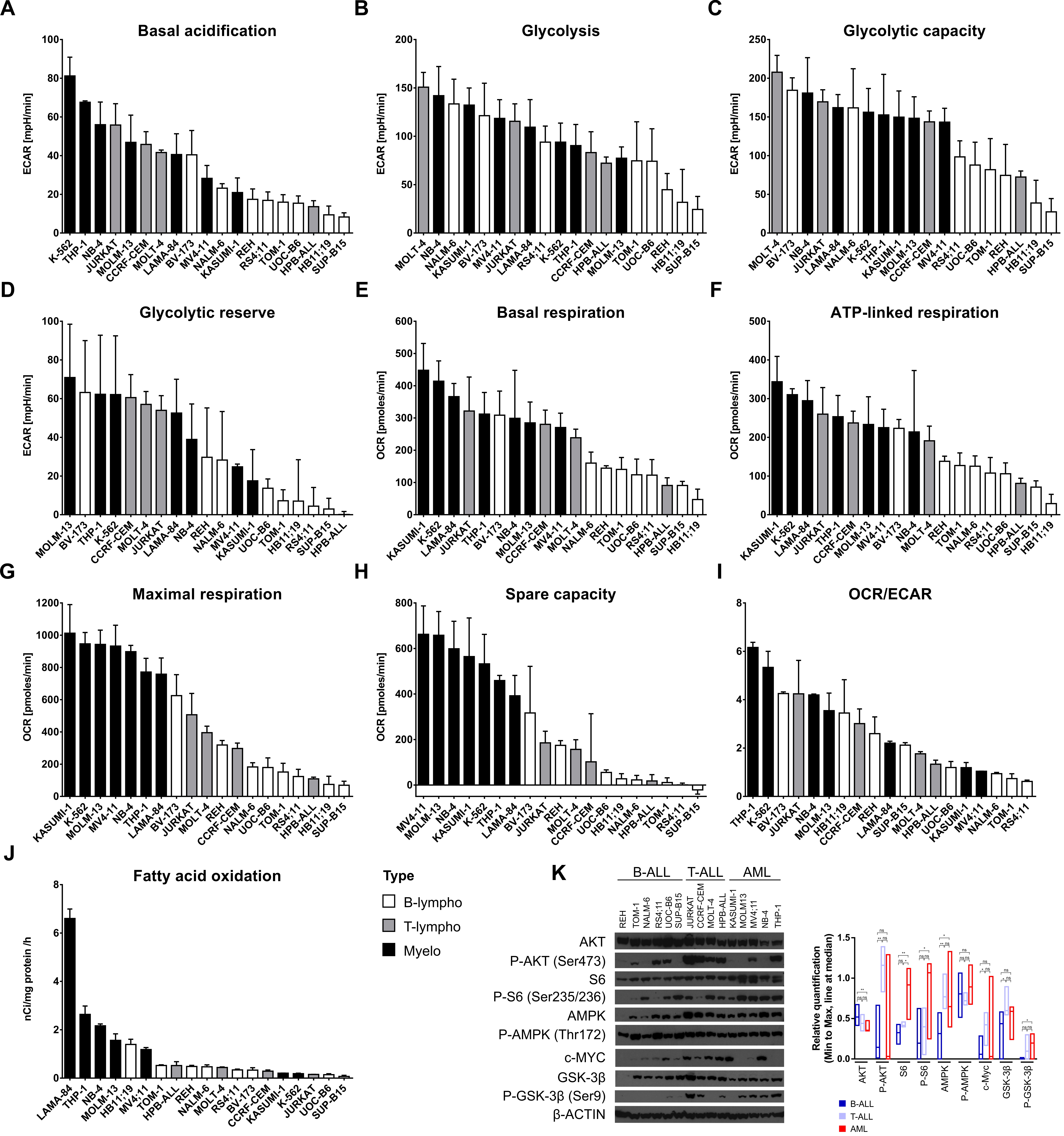

Supplement: Supplementary file 3 — Additional file 3: Supplementary Figure S1. Absolute values of metabolic parameters measured in 19 leukemia cell lines. (A–D) Parameters calculated from glycolytic function. (E–H) Parameters calculated from mitochondrial function. (I) OCR/ECAR ratio calculated after glucose injection during the Glycolysis stress test measurement. (J) FAO rate. AML is presented as black graphs, B-ALL as white and T-ALL as gray graphs. (K) Levels of signaling proteins in the leukemia cell lines were measured by immunoblotting. β-actin was used as a loading control. Relative quantification (normalized to β-actin) was calculated in ImageJ. * p < 0.05, ** p < 0.01, *** p < 0.001. The immunoblot is a representative result of three independent experiments. [file 12885_2020_7020_MOESM3_ESM.jpg]

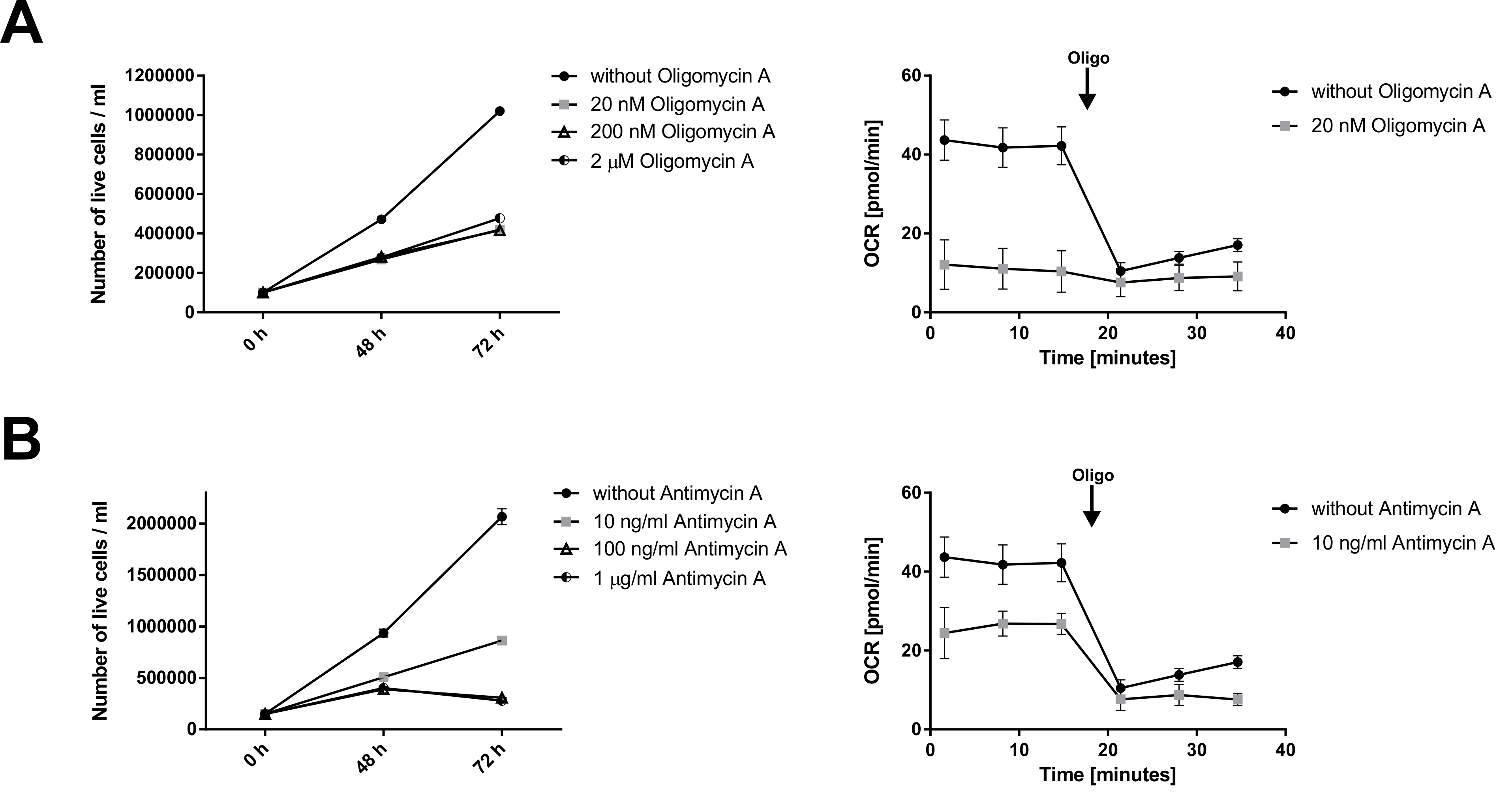

Supplement: Supplementary file 4 — Additional file 4: Supplementary Figure S2. Effect of OXPHOS inhibitors on the growth and mitochondrial respiration of leukemia cell lines. (A) The effect of Oligomycin A (20 nM, 200 nM, 2 μM) on the growth and on the course of mitochondrial respiration of NALM-6 cells. (B) The effect of Antimycin A (10 ng/ml, 100 ng/ml and 1 μg/ml) on the growth and the course of mitochondrial respiration of NALM-6 cells. Cells were counted 48 and 72 h after the treatment. Cell Mito Stress Test was performed after 24 h of treatment. Measurements were done in three biological replicates and the data are presented as mean ± SD. [file 12885_2020_7020_MOESM4_ESM.jpg]

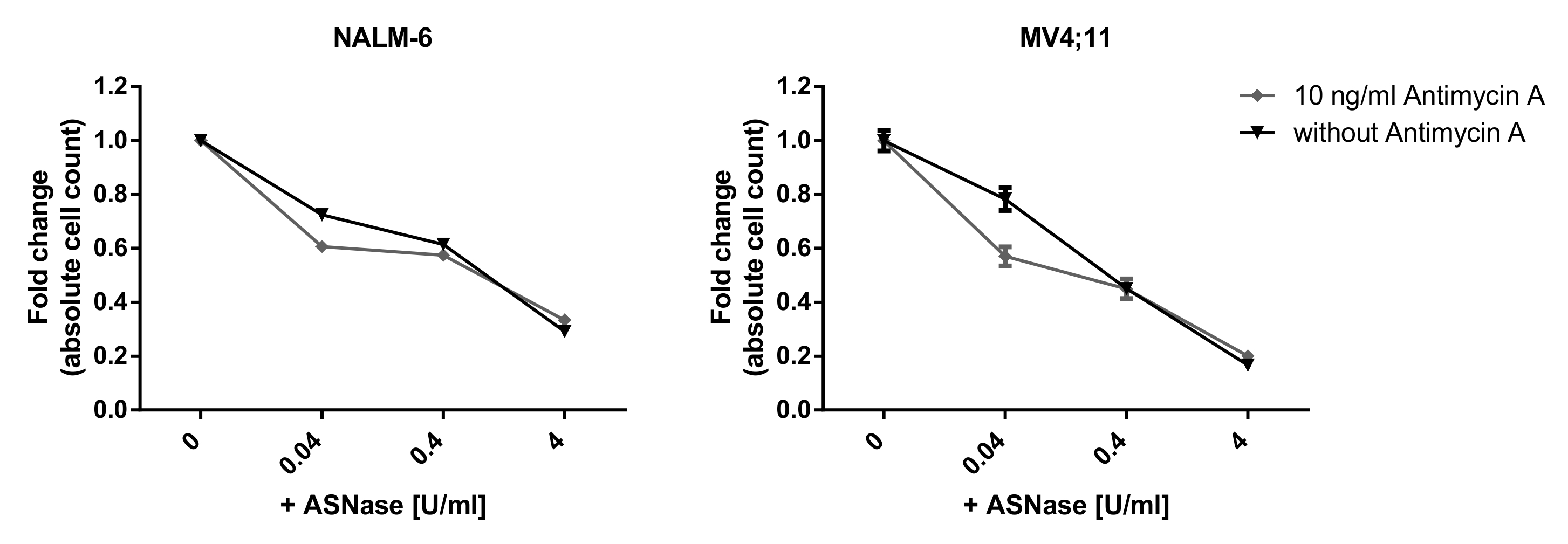

Supplement: Supplementary file 5 — Additional file 5: Supplementary Figure S3. Functional study on the correlation between ETC complex III activity and sensitivity to ASNase. Effect of Antimycin A (10 ng/ml) on the sensitivity of leukemia cell lines (NALM-6, MV4;11) to ASNase. Cells were pretreated with Antimycin A for 1 h or left untreated and then co-treated with ASNase for 48 h. Absolute cell counts were obtained from three independent experiments; data were normalized to untreated controls and are presented as mean ± SD. Measurements were done in three biological replicates and the data are presented as mean ± SD. [file 12885_2020_7020_MOESM5_ESM.jpg]

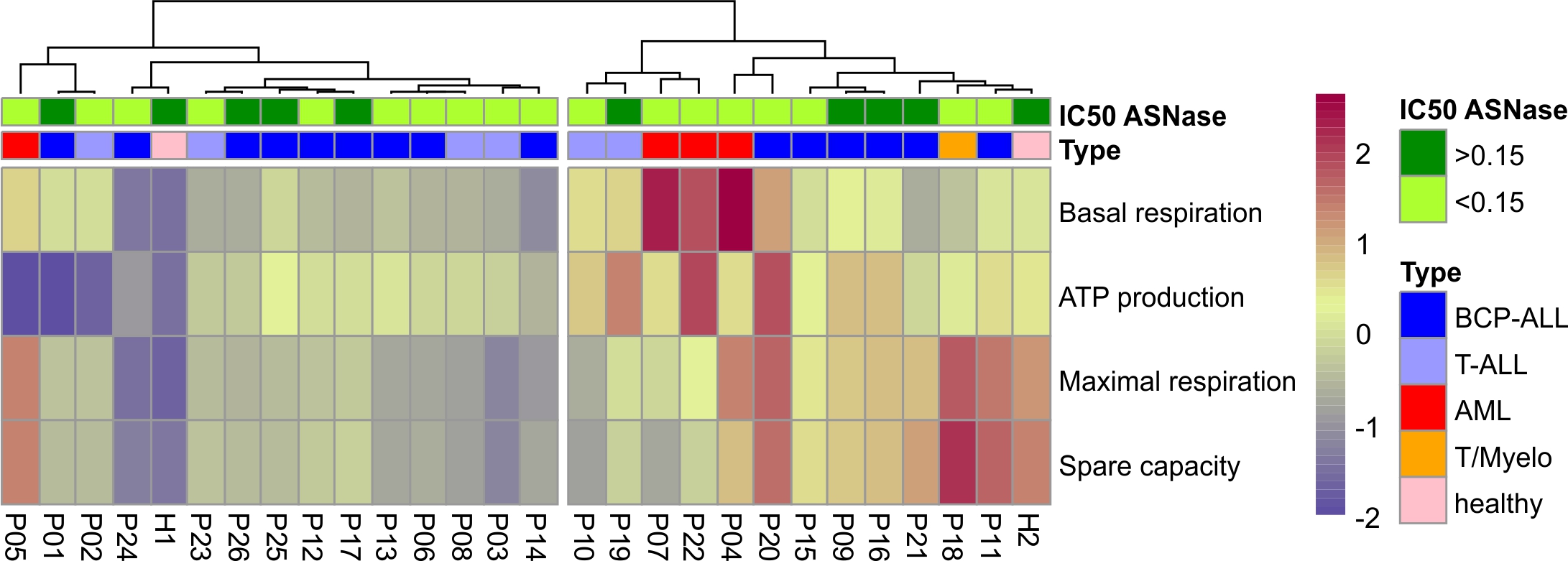

Supplement: Supplementary file 6 — Additional file 6: Supplementary Figure S4. Cluster analysis of patient samples according mitochondrial respiration. Hierarchical cluster analysis of primary leukemia cells and healthy control samples based on parameters calculated from mitochondrial function. Type of leukemia and IC50 ASNase [IU/ml] are indicated for each patient. For more information, see Table 2. [file 12885_2020_7020_MOESM6_ESM.jpg]
